# Long-Term Compressive Strength Development of Steel Fiber Shotcrete from Cores Based on Accelerator Types at Tunnel Site

**DOI:** 10.3390/ma14030580

**Published:** 2021-01-26

**Authors:** Kyong Ku Yun, Seunghak Choi, Taeho Ha, Seong Kwon Kim, Mohammad Shakhawat Hossain, Seungyeon Han

**Affiliations:** 1 Department of Civil Engineering, Kangwon National University, 1 Gangwondaegil, Chuncheon 24341, Korea; kkyun@kangwon.ac.kr (K.K.Y.); donghaebi@kangwon.ac.kr (S.C.); gkxogh10@kangwon.ac.kr (T.H.); 2 Institute for Advanced Construction Materials, Kangwon National University, 1 Gangwondaegil, Chuncheon 24341, Korea; kimskwon@kangwon.ac.kr; 3 KIIT (Kangwon Institute of Inclusive Technology), Kangwon National University, 1 Gangwondaegil, Chuncheon 24341, Korea

**Keywords:** steel fiber shotcrete, long term performance, core compressive strength, accelerators types, cement mineral accelerator, aluminate accelerator, alkali-free accelerators, tunnel site

## Abstract

In this study, cement minerals, aluminates, and alkali-free accelerators incorporated with steel fiber were used to scrutinize the influence of accelerating agents on the long-term performance of tunneling shotcrete. Performance tests were identified based on the core compressive strength of mix shotcrete specimens with different types of accelerating agents throughout timeframes of 1, 3, 6, and 12 months. Here, 37 kg of steel fiber was incorporated into the cement mineral and aluminate mixes, and 40 kg of steel fiber was incorporated in an alkali-free mix for the shotcrete mix design. The KSF 2784 and ASTM 214 standards were followed for specimen fabrication and core cutting. For all specimens, shotcrete test panels of 250 × 600 × 500 mm were manufactured for core compressive strength tests conducted using 100, 75 and 55 mm diameter cylindrical molds and a length-to-diameter ratio of 2. The 1-month compressive strength of all test variables satisfied the Korea Expressway Co. standard of 21 MPa. The core compressive strength of the shotcrete specimens showed a tendency to increase with age, but a strength reduction occurred in 6 months and increased again at 12 months. Moreover, the impact of the diameter changes in the shotcrete core specimens was analyzed based on the mixing. For 12 months, a large increase in the core compressive strength occurred, particularly in the alkali-free specimens. The comparison also focused on the relative strength compared with a cast concrete mold and shotcrete core specimens. It is necessary to use alkali-free accelerators considering the long-term performance of tunnels and worker safety.

## 1. Introduction

Shotcrete is a sprayed concrete with a blend of cementitious material, coarse aggregate, and fine aggregate incorporated with other admixtures through a hose by compressed air [1]. Shotcrete is mainly used for land sliding protection in tunneling and a rapid gain in strength [2]. According to the new Australian tunneling method, shotcrete has been used considerably for subway construction and tunnel construction [3].

Accelerators are mainly used for rapid hardening, the setting of shotcrete, and early gains in strength [4]. Moreover, accelerators are used in shotcrete to reduce rebound and subdue the ground relaxation [5]. In addition, the accelerator agents mainly used in highway tunnels have a high alkali content, which lowers the long-term strength, and this alkaline component is eluted together with the groundwater over a long period of time. When producing high-strength concrete, the accelerators used in the construction field are mineral-based [6]. In addition, the construction performance of shotcrete depends on the use of alkali-free, aluminate-type accelerators [7]. This is because the accelerators enhance the primary buildup thickness and decrease the amounts of rebound and dust. For this reason, it meets the engineering requirements for tunnel engineering. Based on the purpose of the present study, shotcrete accelerators are categorized as aluminate, silicate, alkali-free, and cement-mineral accelerators. According to previous studies, an aluminate accelerator has been used to invigorate the hydration of cement and influence the quick setting [8]. In addition, the alkali-free has less alkaline content, and thus a pH of 0–7, as well as a reduction in the long-term strength effect with a low caustic performance [9,10]. Cement mineral accelerators (C12A7) were used to estimate the performance of the shotcrete of the 28 days compressive and flexural strength with enhanced durability [10,11]. In this research, three types of accelerators were used to determine the mechanical long-term performance of the tunnel shotcrete, and accelerators incorporated with steel fibers were applied for a better shotcrete performance.

According to the material, fibers are classified as glass, metallic, synthetic, and natural fibers. Polyester, polypropylene, and polyethylene are considered synthetic fibers. Natural fibers are extracted from wood cellulose, bamboo, and elephant grass. Moreover, hybrid-fiber-reinforced concrete is another type of fiber added in the construction [12]. According to the ASTM A820/A820M-16 standard, steel fibers are produced using a cold-drawn wire, cut sheet, and melt-extracted, mill cut, and modified cold-drawn wire [13,14]. Song investigated the high strength of steel-fiber-reinforced concrete with different volume fractions of 0.5, 1.0, 1.5, and 2.0%. The author mainly focused on mechanical properties such as the compressive strength, tensile strength, and modulus of a rupture. In addition, the 2.0% volume fraction examined achieved a better performance [15]. Wu et al. examined the three types and shapes of steel fibers with distinct fiber volume percentages that affected the ultra-high-performance concrete mechanical properties [16]. Moreover, the content of the micro steel fiber increases by 0.1 and 0.2%, the compressive strength can also increase at 1, 3,7, 14, and 28 days, respectively [17]. In addition, steel fiber is used to decrease the chloride corrosion effect of ultra-high-performance concrete with different thicknesses, such as 10, 25, and 50 mm [18]. Therefore, steel-fiber-reinforced concrete used in a tunnel construction increases the mechanical properties [19].

In this study, the main purpose was to evaluate the core compressive strength over the long periods of 1, 3, 6, and 12 months, respectively. The core cutting of the enormous amount of concrete work is extremely difficult to accomplish, because it requires a destructive test and extrusion process of the core specimens [20]. For this reason, the ASTM and British standards recommended a minimum core diameter of 100 mm [21]. However, the core diameter is sometimes smaller than 100 mm owing to the narrow space of the reinforcement of the construction, which affects the mechanical properties of the concrete [22,23]. In addition, the maximum size of the aggregate, aggregate types, and length/diameter of the core plays a vital role in the core compressive strength. If these two values increase, the core compressive strength then decreases [24,25]. The correlation factor is not applicable if the normal compressive strength of the concrete reaches up to 40 MPa according to JIS A 1107 and ASTM C42. In addition, if the core diameter is smaller than 100 mm, then the coarse aggregate size must be less than 10 mm. For this reason, different core diameters were used to show the effect of the strength and behavior of the concrete [26,27]. Moreover, as a reference, Ju et al. showed a concrete core compressive strength at 28 days with 30, 50 and 100 mm core diameters. The core compressive strength decreased as the diameter of the specimens decreased. Here, three types of mixtures (MIX) were used. For example, the average core compressive strength test results of MIX-1 were 40.1, 47.3, and 54.3 MPa according to the 30, 50 and 100 mm diameter core specimens. This tendency agrees with the results of previous research. The strength reduction occurs owing to the hydration mechanism and the high-speed rotating drill machine during the core cutting [28]. In addition, Tuncan et al. used 46 and 69 mm diameter cores to measure the core compressive strength. Here, we also scrutinized that the core compressive strength decreased as the diameter decreased. In this study, eight types of mixtures were used for comparing the data at 28 days, and the results showed the same trend. This occurred because of the weaker interface transition zone (ITZ) between the aggregate and cement paste [29]. As per previous studies, it was noted that both the compressive strength of both the concrete and shotcrete core specimens decreased as the size of the specimens decreased, and an extremely high compressive strength was measured in the Φ 100 mm specimen followed by Φ 75 and Φ 55 mm in the concrete and shotcrete specimens, respectively. Therefore, concrete and shotcrete showed the same trend for the core compressive strength and because of the ITZ for the 10 mm aggregate used in all specimens [30,31].

The main purpose of this study was to consider the cement mineral, aluminate, and alkali-free accelerators incorporated with a steel fiber for analyzing the performance of shotcrete in a tunnel project site based on the Korean Expressway Corporation (2003). To evaluate the performance of the shotcrete, we focused on the core compressive strength at 1, 3, 6, and 12 months of all mixes and, here, considered the three samples of each specimens. Furthermore, core size diameters of 100, 75, and 55 mm were considered to satisfy the shotcrete performance. Here, the length and diameter ratio was maintained at two for all cases. Based on the long-term core compressive strength, we identified a better combination of shotcrete mixes. The results show the main novelty of this study.

## 2. Materials and Methods

In this study, three types of accelerators included with the steel fibers’ performance of long-term shotcrete core size compressive strength in the tunnel project site. Shotcrete specimen performance was analyzed by 3 types of core and long-term test process. The performance was then evaluated to determine which diameter is more effective at a tunnel job site based on the long-term performance of the core strength.

### 2.1. Materials

#### 2.1.1. Cement

According to ASTM C150, Portland Cement (Korea) was used in this study. In addition, 61.2% CaO, 20.8% SiO_2_, and 6.3% Al_2_O_3_ were applied in the chemical composition of the cement used, and 3300 cm^3^/g and 3.25 g/cm^3^ were the specific areas and densities, respectively.

#### 2.1.2. Aggregate

Shotcrete aggregate following the Korean Expressway Corporation standard was evaluated in this study. Moreover, 10 mm maximum coarse aggregate, river sand, and crushed fine aggregate sand were utilized in this study. The physical properties of the coarse and fine aggregates used with different accelerator mixtures are shown in Table 1.

#### 2.1.3. Accelerators

Shotcrete used in underground projects is required to meet some basic requirements such as a high early strength and the possibility of being applied in thick layers without risk of a fresh concrete fallout and displacement, particularly in overhead applications. Hence, accelerators are used in shotcrete to develop early strength, reduce the rebound, and suppress the ground relaxation. Accelerators also affect the long-term strength development, durability, and thickness of the shotcrete applied [32]. Over time, the use of accelerators has increased in both dry and wet mix processes. They are mostly used to increase the early strength and reduce the dust and rebound in both the dry-mix process and the wet-mix process to achieve a rapid setting and early strength. Their effect on early strength development is mainly determined by their chemical base, dosage, chemical composition of the binder, presence of mineral additions, and temperature [33]. The chemicals in the accelerating admixtures influence the rate of cement hydration, which results in a shortened setting time and increased rate of early strength development [34]. In this study, cement mineral, aluminate, and Korean-made alkali-free accelerators were used. Table 2 shows the physical properties of the accelerators.

#### 2.1.4. Steel Fibers 

The use of fibers to strengthen the materials, which are much weaker in terms of tension than in compression, dates back to ancient times where straws were used to reinforce clay bricks [35]. Five types of metallic fibers, mainly steel fibers, are discussed in the ASTM standard based on the product or process used for production, i.e., cold-drawn, cut sheet, melt-extracted, mill cut, and modified cold-drawn wires [36]. In this study, a 35 mm hook type of steel fiber was used. The physical properties of the steel fiber depend on the aspect ratio and tensile strength. Here, each mix design used steel fibers with different physical properties, such as the cement mineral, aluminate, and alkali-free mixed steel fibers with aspect ratios of 62.0, 60.1, and 60.9 at 1123, 1043, and 977 N/mm^2^, respectively.

### 2.2. Mix Design

The shotcrete mix design was mainly made by the Korean Expressway Co. (2003), which follows the guidelines for tunnel shotcrete quality and standard shotcrete composition. Five percent cement minerals (CM), 5% aluminate (AL), and 7% alkali-free (AF) were set based on the usual mixture trends used by the experts at the construction site. Moreover, for the sustainable slump and air content, a high-range water reduction agent was used in the mixture design in this study. Mix design is shown in Table 3.

### 2.3. Specimen Preparation 

Concrete and shotcrete specimens were manufactured on the tunnel project site for test purposes. Concrete was produced using a ready-mix plant and concrete mixer truck. By contrast, the shotcrete test was accomplished in situ; a batch plant was used to mix the ingredients, and the mixed concrete was carried to the shotcrete pumping machine by a ready-mixed concrete truck. The CO used for the concrete mold and the SH for the shotcrete core specimen are identified by their symbol. Moreover, every mix had 3 samples for performing the test of the specimens.

**Figure 1:**
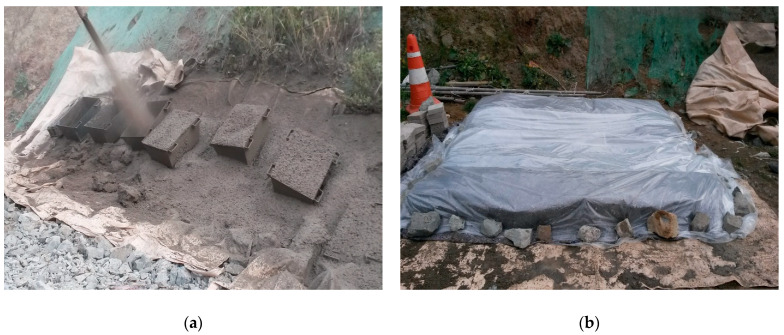
Test sample preparation, (**a**) Shooting at test panels, (**b**) Sample curing.

In this research, CO specimens were produced at the project site incorporating steel-reinforced concrete, which was carried from the batching plant by a ready-mixed concrete truck. These specimens did not include accelerators. However, SH specimens were manufactured at the job site with steel fiber reinforced concrete and accelerators and transferred from the batching plant using a ready-mixed concrete truck. Subsequently, SH was shot to the shotcrete test panels of 250 × 600 × 500 mm for the core compressive strength Figure 1a. For the core compressive strength test, core cutting was used to maintain the diameter of the core at 100, 75, and 55 mm. The process follows the KSF 2784 standard. The length-to-diameter (L/D) ratio of the core specimens was maintained according to ASTM C42-90 and the British Standard Institute (BIS) for both CO and SH. In this study, L/D was 2, which was fixed for all specimens. All specimens were cured in natural, which is shown in Figure 1b. This test was conducted for 1, 3, 6, and 12 months, and for the core compressive test, core cutting occurred on the morning of the test day, and specimens were prepared during the afternoon of the test. Figure 1 shows the preparation of the test sample specimens.

### 2.4. Core Compressive Strength Test

**Figure 2:**
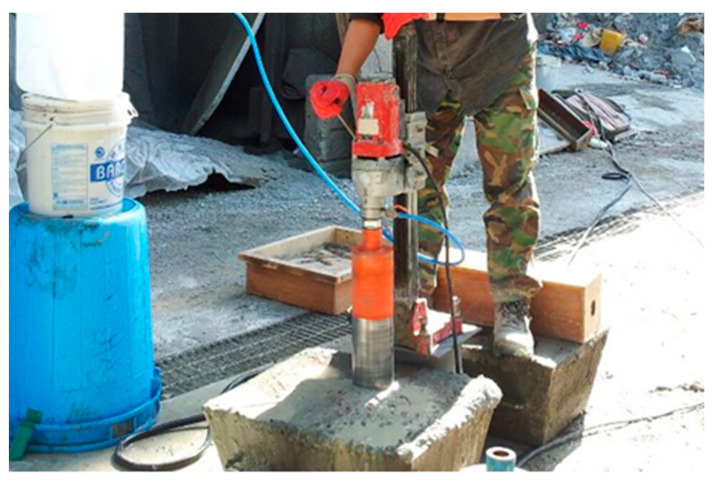
Core cutting from test panel.

The compressive strength of the concrete was tested using the KSF 2405 standard test method. In this study, cylindrical core specimens were drilled from the concrete and shotcrete test panels of 250 × 600 × 500 mm in size to obtain diameters of 100, 75, and 55 mm to conduct the core compressive strength test shown in Figure 2. This test was performed at 1, 3, 6, and 12 months of age with L/D (2) cylindrical specimens to determine the compressive strength of the mix for the specified ages by considering three specimens for each mix. The properly cured specimens were prepared for the test by leveling and smoothing both end surfaces of the specimens to apply the load uniformly. The compressive strength of the specimen was calculated by dividing the maximum load carried by the specimen by the average cross-sectional area.

## 3. Results and Analysis 

### 3.1. Effects of Accelerators on the Core Compressive Strength Test Result

#### 3.1.1. One-Month Core Compressive Strength Result

**Figure 3:**
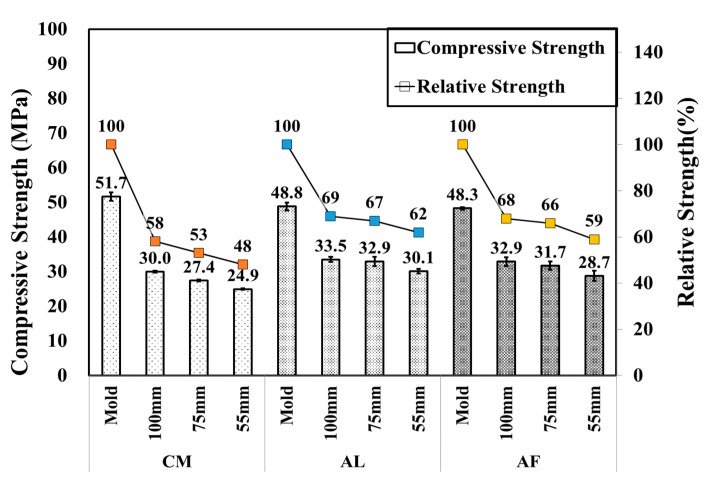
Core compressive and relative strengths of CO and SH specimens by accelerators for 1 month-old specimens.

**Figure 4:**
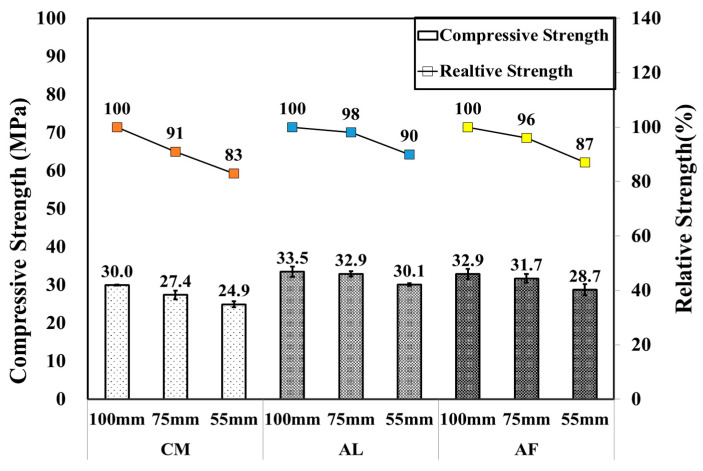
Core compressive and relative strengths of SH specimens by accelerators for 1 month-old specimens.

SH specimens were mainly used for the core compressive strength test, which was conducted by drilling 100, 75, and 55 mm diameter specimens from the shotcrete box specimens to compare the strength by the size of the specimens. Moreover, one extra CO specimen was produced to compare the result with that of SH. It was observed that the compressive strength of the SH specimen decreased as the size of the specimens decreased, and an extremely high compressive strength was measured in the ø 100 mm specimen followed by the ø75 and ø55 mm SH specimens, similar to the CM mixtures of the SH core with a compressive strength of 30.0, 27.4, and 24.9 MPa, respectively. Here, CO is the mold compressive strength of 51.7, 48.8, and 48.3 MPa of CM, AL, and AF, respectively. Figure 3 shows the CO mold compressive strength and SH core compressive strength test results of the specimens compared by core size. Moreover, Figure 4 shows the relative strengths of the CO mold and SH core, where the mold strength is considered to be 100%, and measured the others’ SH core size relative strength, respectively. In addition, error bars are added to Figure 3 and Figure 4.

#### 3.1.2. Three-Month-Old Core Compressive Strength Test Result

**Figure 5:**
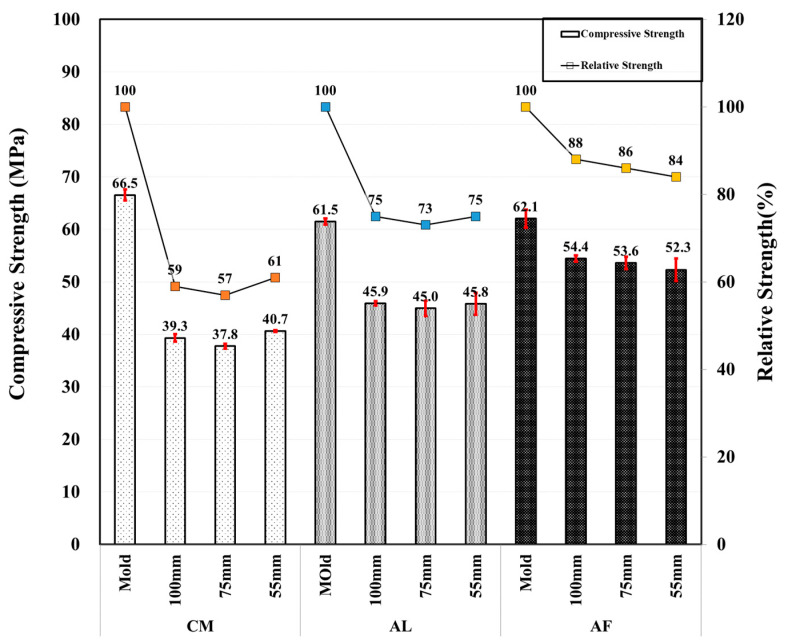
Core compressive and relative strengths of CO and SH specimens by accelerators for 3 month old specimens.

**Figure 6:**
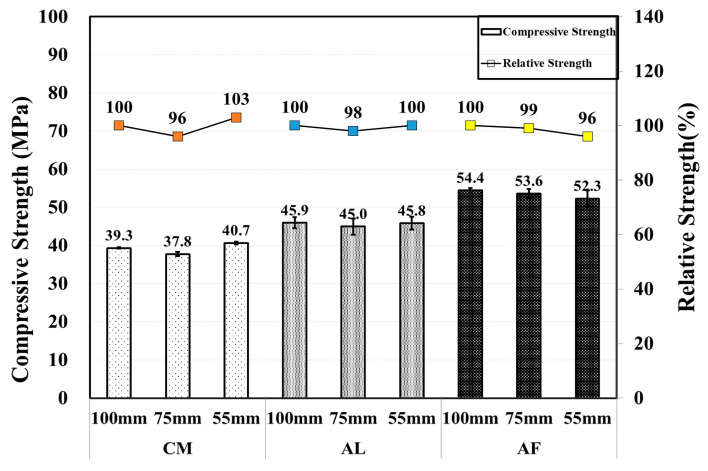
Core compressive and relative strength of SH specimens by accelerators for 3 month-old specimens.

Unlike after the first month, in the compressive strength test result, where there was a significant strength reduction with a reduction in the specimen core size, a relatively higher compressive strength was measured in the Φ 55 mm specimens, except for the AF mix. The compressive strength of the AF mixture is 54.4, 53.6, and 52.3 MPa with diameters of 100, 75, and 55 mm, respectively, which were higher than those of the AL and CM mix. However, the Φ 55 mm specimens of SH were higher than those of other diameter specimens. The AF relative strength of the SH specimens were 88, 86, and 84% in the Φ 100, Φ 75, and Φ 55 mm core specimens, respectively. Figure 5 shows the comparison of the compressive strengths of the CO and SH core specimens, and a comparison between the core specimens are shown in Figure 6. Here, the error bars are also shown in the following figure.

#### 3.1.3. Six-Month Core Compressive Strength Test Result

**Figure 7:**
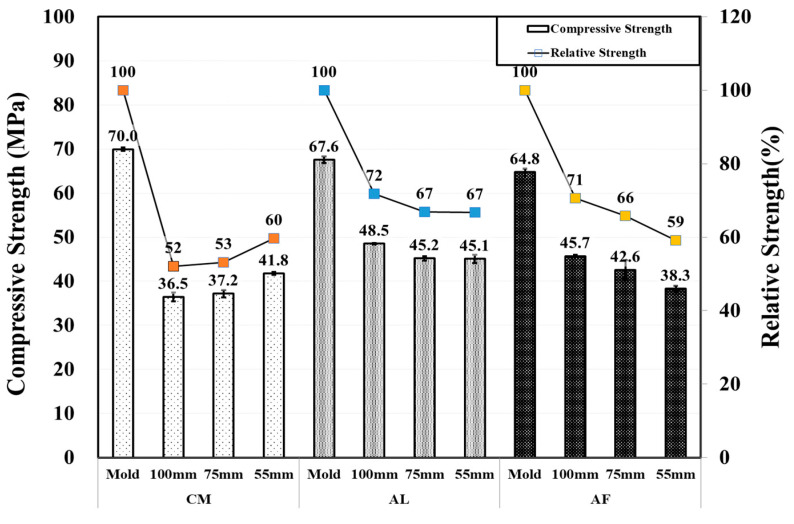
Core compressive and relative strengths of CO and SH specimens by accelerators for 6 month old specimens.

**Figure 8:**
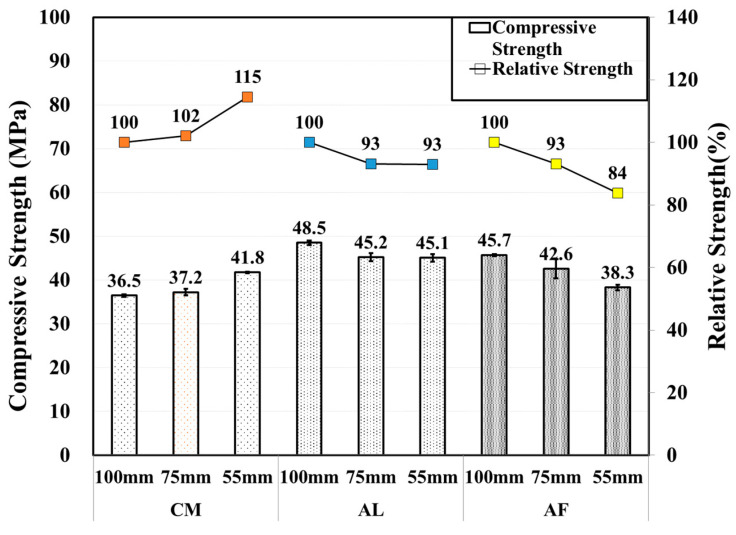
Core compressive and relative strengths of SH specimens by accelerators for 6 month-old specimens.

In this test, the AL and AF, SH core specimen’s compressive strength, decreased with the decrease in core size as expected. On the contrary, in the CM mix compressive strength was observed to increase as the specimen’s core size decreased 41.8 MPa in Φ 55 mm, there was also high strength reduction compared with the CO specimen. Compared to the 3-month test result, the AL mix specimens showed an increase in strength, while there was a strength reduction in the AF mix specimens and in the CM Φ 100 mm and Φ 75 mm specimens. The AF mix Φ 55 mm specimen showed the highest 27% strength reduction, while the CM mix Φ 55 mm specimen’s compressive strength increased by approximately 3%. Figure 7 and Figure 8 show the comparison of the CO and SH core specimen’s compressive strength and the compressive strength of the SH core specimens by the core size with standard error bars.

#### 3.1.4. Twelve-Month Core Compressive Strength Test Result

**Figure 9:**
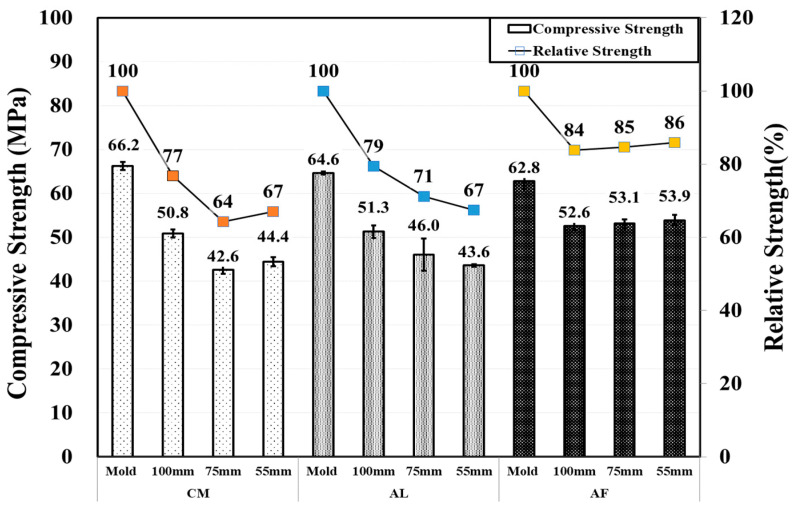
Core compressive and relative strengths of CO and SH specimens by accelerators for 12 month old specimens.

**Figure 10:**
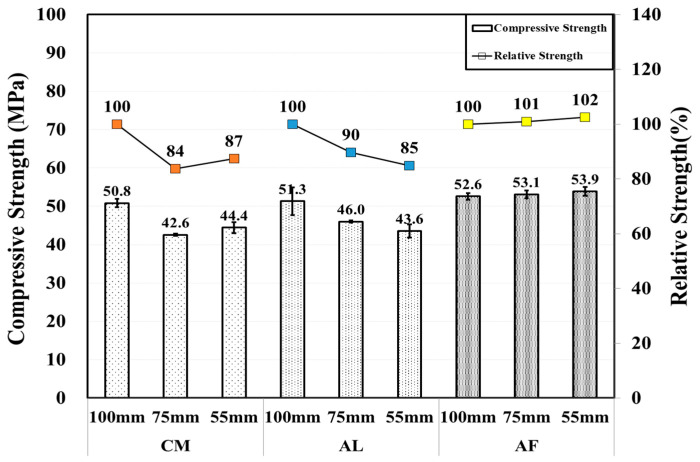
Core compressive and relative strengths of SH specimens by accelerators for 12 month old specimens.

The SH core compressive strength of the AL mix was observed to decrease with a decrease in core size of the specimen. By contrast, the core compressive strength of the AL mix showed a tendency to slightly increase as the core size decreased. In the CM mix, the Φ 75 mm specimen showed a lower strength, and a higher core compressive strength was measured in the Φ 100 mm specimen. Compared with the 6-month test result, there was a significant increase in the compressive strength in all accelerator mixes except the aluminate mix of the ø55 mm specimen, which showed a slight strength reduction. In particular, the AF mix ø55 mm specimen showed an approximate increase in strength of 41%. Figure 9 shows the compressive strength of the CO and SH core specimens, and Figure 10 shows the core compressive strength by the diameter of the specimens with error bars.

### 3.2. Core Comprehensive Review of Shotcrete Long-Term Performance Test Results

In this section, the analysis of the 1 to 12 month old core compressive strength test results for each accelerator mix incorporated with steel fiber are discussed in terms of the long-term shotcrete performance.

**Figure 11:**
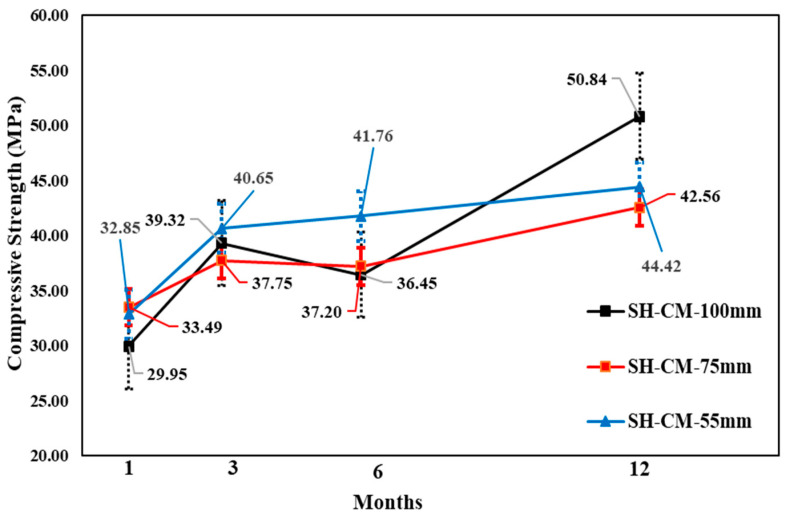
Core compressive strength of cement mineral accelerator mixture by age.

**Figure 12:**
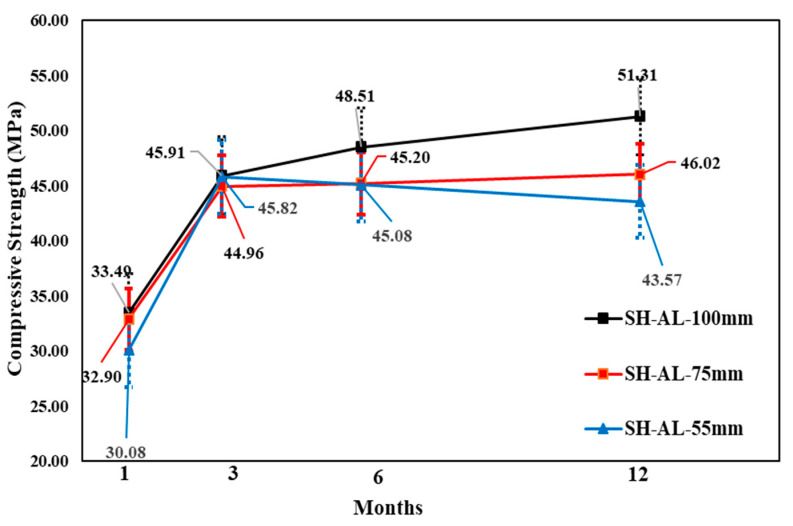
Core compressive strength of aluminate accelerator mix by age.

**Figure 13:**
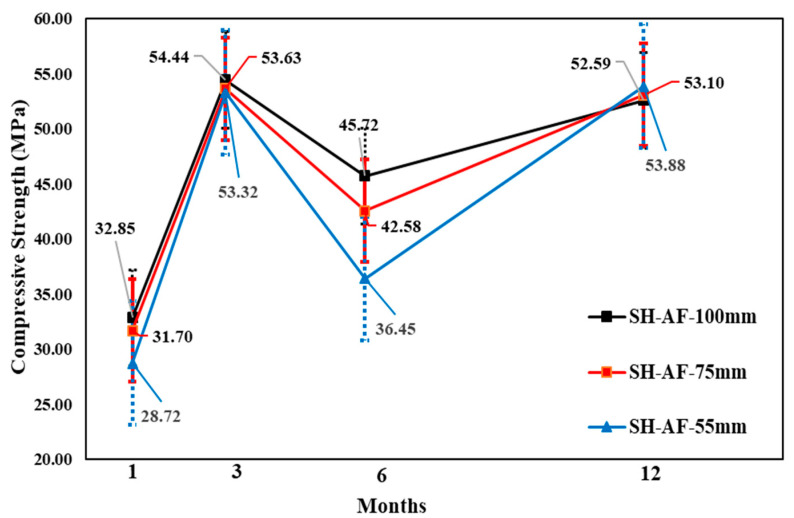
Compressive strength of alkali-free accelerator mix core by age.

The change in core compressive strength from the 1 to 12-month-old specimens showed that, for all accelerators, the SH specimen gains strength for up to 3 months, followed by a relatively small strength reduction of 6 months, and another increase at 12 months. The AF mix showed the highest strength reduction of 6 months based on the 3 month result, although the core compressive strength values were higher than the others for core diameters of 100, 75, and 55 mm, respectively. By contrast, different behaviors were observed in the CM mix for Φ 55 mm specimens, because the core compressive strength was higher (41.76 MPa) than the other values. Here, the changes in the core strength of the AF mixture were not steady; however, in the long term, the highest core compressive strength was shown. In addition, the AF mixture in the Φ 55 mm SH specimens showed a slightly higher strength than the Φ 75 mm specimens at 12 months. Moreover, the CM mixture specimens showed the same behavior as that of the AF mixture specimens. Figure 11, Figure 12 and Figure 13 show the change in compressive strength by age in each accelerator mix based on the findings of the test.

## 4. Conclusions

In this study, an accelerating agent incorporating steel fibers was used to measure the long-term shotcrete performance. A summary of the results of this study is described in the following.

1. Three types of accelerating agents were used for experimental purposes: CM, AL, and AF. Steel fiber (37 kg) was incorporated into the CM and AL mix, and 40 kg of steel fiber was incorporated into the AF mix. Moreover, 1, 3, 6, and 12 month spec mens were applied to evaluate the long-term performance of shotcrete at a tunnel site.
2. This study mainly focused on the core compressive strength for identifying the performance of the shotcrete. As a result, the CO (Φ100 mm) and SH specimens with Φ 100, Φ 75, and Φ 55 mm diameters with a length/diameter ratio of 2 in all specimens was shown.

In the 1 month compressive strength test, the Korea Expressway Co. standard of 21 MPa was satisfied for all test variables. Here, the core compressive strength test results showed that the CO specimens have higher strength values than the SH specimen strength values for all types of mixing accelerators.

Moreover, the SH specimens showed increasing criteria for the core compressive strength of all specimens at up to 3 months, which drastically decreased in the 6-month test results of the AF and CM mixed specimens. Then, another increase at 12 months was shown in the test results of the AF and CM mixed specimens, although the core compressive strength of the AL mixed specimens increased gradually with respect to time except for the ø55 mm specimens in the 6- and 12-month test results at 45.08 and 43.57 MPa, respectively. Here, the AF mix specimens test results at 12 months were 52.6, 53.1, and 53.9 MPa based on the Φ 100, Φ 75, and Φ 55 mm, respectively, which are lower than the 3-month core compressive strength. This behavior indicates that AF reduces the long-term shotcrete performance. By contrast, the CM and AL mix specimens increased the long-term shotcrete performance. As in previous research, if the core size decreases, the core compressive strength also decreases. Here, the core compressive strength of the SH specimens showed the same behavior at the 1-month results. However, the CM specimens showed a different behavior, with core compressive strengths of 39.3, 37.8, and 40.7 MPa at Φ 100, Φ 75, and Φ 55 mm, respectively, at 3 months. In addition, the AF mixed specimens showed the highest core compressive strengths of 52.6, 53.1, and 53.9 MPa based on Φ 100, Φ 75, and Φ 55 mm diameters, respectively, at 12 months.

Thus, based on the results for each diameter, long-term changes in behavior do not match those of previous studies, which is the main finding and novelty of this study. In addition, the relative strengths of the AF mixed Φ 75 and Φ 55 mm specimens are 101% and 102%, respectively. Here, Φ 100 mm core specimens are considered to have a 100% relative strength. In addition, the AF accelerator is safer than the AL accelerator. The AF is safe for the environment and workers because it has no alkali content. Therefore, based on all criteria, the AF is more useful than the AL and CM for tunnel shotcrete.

In conclusion, it was identified that the AF mixed specimens incorporated with steel fiber are more effective for the tunnel project. In addition, core cutting in the construction affects the compressive strength owing to the ITZ, but the AF mix specimens showed the highest core compressive strength test results beyond the AL and CM mix specimens from a long-term perspective. In future studies, more tests will be conducted, such as on the flexural toughness, and changes in the chemical composition in the concrete from hydration will be determined to find an appropriate combination of parameters.

## Figures and Tables

**Table 1:** Physical properties of fine and coarse aggregates.

Test	Aggregate	Density (g/cm^3^)	Fineness Modulus	Division	Standard
Cement mineral mix	Fine	2.61	3.86	Aggregate for shotcrete	Mixed aggregate
	Coarse	2.70			
Aluminate mix	Fine	2.61	3.84		
	Coarse	2.70			
Alkali mix	Fine	2.61	3.78		
	Coarse	2.70			

**Table 2:** Physical properties of the accelerators.

Accelerator	Type	Specific Gravity	pH	Solid Content	Chemical Components	Function
Cement mineral	Powder	2.76	10–12	99.2	C_12_A_7_	Easily combined in shotcrete with water
Aluminate	Liquid	1.45	13 ± 2	45.7	NaAlO_2_ and KAlO_2_	High early strength
Alkali-free	Liquid	1.36	2.6	42.0	(%Na_2_O + 0.658 ∗ %K_2_O	Avoid skin burns, loss of eyesight, respiratory health problems, reduce rebound, strength gain

**Table 3:** Shotcrete performance test mix design.

Accelerator	Gmax (mm)	Slump (mm)	W/C	S/a (%)	Unit Content (kg/m^3^)					
					Water	Cement	Sand	Gravel	High-Range Water-Reducing Agent	Steel Fiber (kg/m^3^)
CM	10	100	0.44	65	210	480	1047	568	4.80 (1.0%)	37
AL			0.43	64.7	213	492	1074	608	3.936(0.8%)	37
AF			0.44	62.1	206	465	1011	622	4.65 (1.0%)	40

## Data Availability

Not applicable.
